# The Views of People With Intellectual Disabilities About What Contributes Towards Optimal End‐of‐Life Care: A Qualitative Evidence Synthesis

**DOI:** 10.1111/jar.70067

**Published:** 2025-06-11

**Authors:** Corrina Alex Bebbington, Elizabeth Croot

**Affiliations:** ^1^ Sheffield Centre for Health and Related Research, University of Sheffield Sheffield UK

**Keywords:** end‐of‐life care, inequalities, intellectual disabilities, literature review, palliative care, qualitative evidence synthesis

## Abstract

**Background:**

People with intellectual disabilities face inequities in access to end‐of‐life care and inequalities in its quality and delivery. This review aimed to synthesise qualitative evidence to understand their own perspectives about what contributes to optimal end‐of‐life care.

**Methodology:**

Data from 93 participants in five qualitative studies were thematically synthesised to identify optimal care and inform recommendations.

**Results:**

Four overarching and interrelated analytical themes were generated. (1) Optimal care recognises heterogeneity and is person‐centred. It aligns with individuals' wishes and preferences which are established through ‘active’ communication. (2) This enables an individual's holistic support needs to be identified. (3) It fulfils ethical obligations around autonomy, equity and a person's ‘right to know’. (4) It involves the necessary people to ensure all needs are met.

**Conclusion:**

Optimal end‐of‐life care is person‐centred, holistic, uses ‘active’ communication, meets ethical obligations and involves the necessary people in care.

**Summary**

- People with intellectual disabilities do not always get the same quality of end‐of‐life care as people without intellectual disabilities.
- This review found five papers which looked at how end‐of‐life care should be delivered from the point of view of people with intellectual disabilities. We used these papers to explain what good end‐of‐life care looks like for people with intellectual disabilities.
- Good end‐of‐life care is:
  ○ Person‐centred: This means talking and listening to the person to find out what is important to them and how they want to be cared for.
  ○ Holistic: This means thinking about and caring for all areas of a person's life.
  ○ Ethical: This means treating people in the right way, respecting their choices and making sure they get fair treatment.
  ○ Involves the necessary people: this means making sure all the people who need to be involved are included in caring for the person at the end of their life.

## 1 Introduction

People with intellectual disabilities are now living longer due to improvements in health and social care, and are dying of similar life‐limiting conditions as the general population (Cithambaram et al. 2020; Patja et al. 2000; Tuffrey‐Wijne et al. 2016; Voss et al. 2017). However, life expectancy is still between 14 and 17 years lower compared with the general population (The Learning Disabilities Mortality Review Programme 2019). These gaps are underpinned by inequalities across the wider determinants of health, and this includes inequities in access to end‐of‐life care and inequities with its delivery.

There is no international consensus in the literature around palliative and end‐of‐life care definitions and associated timeframes. These terms are sometimes used synonymously, especially in the UK and North America (European Association for Palliative Care 2015). This has hampered comparisons and the development of standards (European Association for Palliative Care 2015). Despite these challenges, there are examples of position statements, norms and standards around palliative care across continents, and in over 30 countries (African Palliative Care Association 2011; American Association on Intellectual and Developmental Disabilties 2020; European Association for Palliative Care 2015).

Internationally, palliative care is underdeveloped, and accessing quality care is rare outside higher‐income countries (Worldwide Palliative Care Alliance 2014). In 2014, at the World Health Assembly, palliative care was identified as a core component of health systems, and member states were called upon to improve access. Palliative care is also considered part of universal health coverage, and ensuring access to care and pain relief are both ethical and human rights obligations. The World Health Organisation identifies domains for palliative care which can be used as a framework for measuring care quality. These domains are around: structure and process; holistic aspects of care; care of the imminently dying person; and ethico‐legal aspects (World Health Organisation 2016).

In the UK, there appears to be consensus that ‘end‐of‐life care’ refers to the last year of a person's life (NHS England 2022). A number of national reports have reviewed priorities (NHS Benchmarking Network 2019, 2020), ambitions (Palliative Care For People With Learning Disabilities Network 2017) and provided guidance (National End of Life Care Programme 2017) around end‐of‐life care, some of which are specific to people with intellectual disabilities. However, concerns remain in both the research literature (Cithambarm et al. 2021; Heslop et al. 2014; Rickard and Donkin 2018; Tuffrey‐Wijne et al. 2016) and reviews of practice (Care Quality Commission 2016; The Learning Disabilities Mortality Review [LeDeR] Programme 2000), about access to end‐of‐life care, including referral to specialist services, and inequities in service quality and delivery. The UK Health Equality Act (Equality Act 2010) mandates that public sector organisations must provide equitable services to people with protected characteristics; this includes making reasonable adjustments around the delivery of end‐of‐life care for people with intellectual disabilities. There is equivalent legislation internationally, for example, the Netherlands (Equal Treatment Law of the Kingdom of the Netherlands [European Commission 1994]). Therefore, there is a need to ensure that end‐of‐life care is equitable for people with intellectual disabilities.

Currently, the majority of the literature around the end‐of‐life care of people with intellectual disabilities has been from the perspectives of professionals or supporters (Arrey et al. 2019; Bekkema et al. 2014a, 2014b; Ryan et al. 2010, 2011; Wagemans et al. 2013). It is rarer to find research from the perspective of people with intellectual disabilities (Bekkema et al. 2016; Cithambarm et al. 2021). This is despite evidence to suggest that these perspectives differ, and that people with intellectual disabilities are both able and willing to talk about death and dying (Cithambarm et al. 2021; Koch et al. 2015; Schmidt et al. 2010; Scott and Havercamp 2018; Tuffrey‐Wijne et al. 2013). Correspondingly, there has been no synthesis of evidence exploring what people with intellectual disabilities consider to contribute towards optimal end‐of‐life care, as confirmed with the International prospective register of systematic reviews (PROSPERO) and the Cochrane Library. This review aimed to address this gap in knowledge by synthesising relevant qualitative evidence from all settings from the perspective of people with intellectual disabilities.

## 2 Methodology

### 2.1 Study Design

This qualitative evidence synthesis identified, appraised and synthesised research literature from the perspectives of people with intellectual disabilities around end‐of‐life care. Initial scoping of the literature revealed that this topic is under‐researched, and perspectives of people with intellectual disabilities are under‐explored. Thematic synthesis methodology (Thomas and Harden 2008) was chosen as a well suited method for synthesising conceptually thin data, because it goes beyond the findings of primary studies to generate new explanations, and is therefore less dependent on the underpinnings of the primary studies when compared with alternative methodologies (Barnett‐Page and Thomas 2009; Booth et al. 2016; Gough et al. 2012; Thomas and Harden 2008). Furthermore, the output of thematic synthesis methodology is appropriate to both practitioners and policy‐makers (Thomas and Harden 2008). The Enhancing transparency in reporting the synthesis of qualitative research (ENTREQ) checklist was used in the reporting of this study (Tong et al. 2012).

### 2.2 Search Strategy and Data Sources

The search strategy was pre‐planned, systematic and sought to find all available reports.

Search terms were informed by initial literature scoping, and were identified for the main components of the research question, adapting the ‘SPICE’ framework (Booth 2006): Setting, Perspective, Intervention and Environment, there was no Comparator. Medical Subject Headings (MeSH) and free‐text terms were used, with relevant search operators and wildcards adjusted for each database. Search terms were refined iteratively based on piloting and feedback from two experienced information specialists to ensure the sensitivity of the strategy for returning relevant records. Synonyms were included for both palliative and end‐of‐life care, due to discrepancies in terminology internationally (Tuffrey‐Wijne et al. 2016). Subsequent screening was used to differentiate between these terms, and the ‘optimal’ nature of care. An example search strategy is shown in Appendix A.

PsycInfo, MEDLINE, Embase and CINAHL were searched systematically, from 2011 until July 2022. The searches were limited by language (English), study design (qualitative) and date (published during or after 2011 due to the introduction of the UK Equality Act in 2010).

Additional search techniques were supplementary to database searching, and included citation and reference list searching, and contacting a topic‐specific academic expert.

### 2.3 Study Screening and Eligibility Criteria

Records identified from databases and other methods were imported into ‘Zotero’ reference management software (Takats et al. 2023) and duplicates were removed. Records were screened by their titles and abstracts then, full‐text reports were reviewed using the inclusion and exclusion criteria in Table 1. To minimise bias with this process and ensure sensitivity in returning relevant records, a second reviewer independently reviewed a proportion (20%) of the full‐text reports. Both reviewers discussed any inconsistencies in study screening until consensus was reached. There were challenges with differentiating between end‐of‐life care and palliative care during study screening; decisions were based on the framing of these terms by study authors, and being guided by ‘last year of life’ as an accepted definition of end‐of‐life care in the UK (NHS England 2022).

**TABLE 1:** Eligibility criteria for the inclusion of studies in the final QES.

SPICE component of the research question and limits	Inclusion criteria	Exclusion criteria
(S) Setting	All settings including primary, secondary and informal care settings. All geographies.	n/a
(P) Perspective	People with intellectual disabilities only.	People solely diagnosed with other conditions that do not affect intellectual disability, e.g., dyslexia, autism and dementia.
	People with co‐morbid conditions which would either underlie a diagnosis of intellectual disability or represent a life‐limiting condition for a person with an intellectual disability.	Proxy perspectives for people with intellectual disabilities.
		Studies were excluded if the perspectives of people with intellectual disabilities could not be distinguished from others in their reporting
(I) Interest (phenomenon of) (1)	End‐of‐life care.	Palliative care, advanced care planning or other related phenomenon more generally without an explicit focus on end‐of‐life care. Studies were excluded if the phenomenon of interest could not be differentiated and thus extracted clearly from other phenomena, such as palliative care more broadly
(I) Interest (phenomenon of) (2)	Studies which explored what contributes to optimal end‐of‐life care.	n/a
Environment/time	The end‐of‐life care phase only.	The palliative care phase more broadly.
Study type	Qualitative studies only.	All other study design types and other types of research literature such as discussion papers, opinion papers, conference abstracts, editorials, letters, comments and guidelines. Mixed‐methods studies where qualitative data could not be extracted.
Date	2011 onwards	n/a

### 2.4 Quality Appraisal Items and Process

The Critical Appraisal Skills Programme (CASP) qualitative appraisal checklist (Critical Appraisal Skills Programme 2018) was used to appraise studies for their trustworthiness and robustness by assessing the quality of their reporting. There is consensus in the literature that the results of quality appraisal should inform the subsequent data synthesis. There are several strategies for implementing this: excluding ‘low’ quality studies, weighting the data synthesis based on quality, or using sensitivity analyses (Carroll et al. 2012; Carroll and Booth 2015; Long et al. 2020). A sensitivity analysis was chosen as the most risk‐adverse option, meaning that no study was lost to the data synthesis. The sensitivity analysis was conducted using an approach described by Carroll et al. (Carroll et al. 2012) (see Appendix B). The results of the sensitivity analysis determined if any of the included studies were privileged based on their reporting quality (Carroll et al. 2012; Franzel et al. 2013; Long et al. 2020).

### 2.5 Data Extraction and Synthesis

Data were extracted by CB, using an adapted qualitative data extraction form (Johnson 2016), around the following parameters: funding, declarations of interest, participant demographics, headline findings, research question(s), aims, setting and methods, severity of intellectual disability and eligibility criteria (for recruitment). All first‐ and second‐order textual data under the ‘results’ headings of included studies were extracted. Data were stored and managed using NVIVO software.

Extracted data were thematically synthesised (Thomas and Harden 2008) by CB. Firstly, an inductive approach was used to code textual data line‐by‐line as ‘free’ codes within NVIVO. Subsequent studies were coded using existing codes, with additional codes created where applicable. LC reviewed the coding for one included study to check that the use of codes was appropriate and consistent. Codes and code definitions were discussed and refined between reviewers before CB coded the remaining studies. The codes were compared and organised into a hierarchal structure of descriptive themes and related subthemes. Finally, the themes were examined in relation to the original research question to develop analytical themes and identify recommendations.

## 3 Results

### 3.1 Study Selection

**FIGURE 1:**
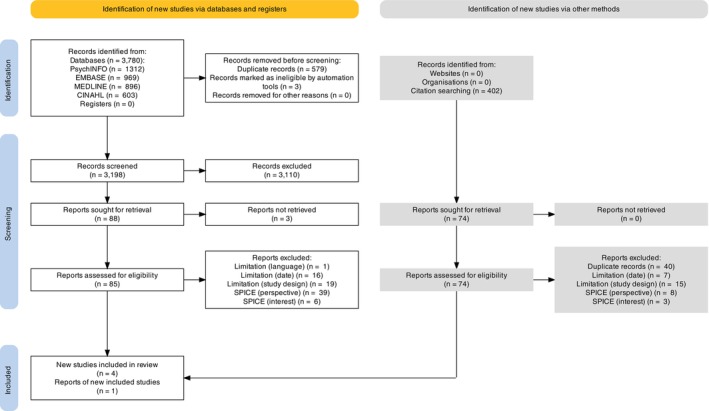
A PRISMA flow diagram showing the results of study screening.

The study selection results are outlined in Figure 1, with reasons for the exclusion of reports at full‐text stage detailed. This resulted in five papers for inclusion in the final qualitative evidence synthesis.

### 3.2 Study Characteristics

There were similarities in the five included studies relating to: research setting, participant demographics, recruitment strategy, data collection and analysis methods. Differences between studies related to: participant numbers, the phenomena of interest and headline findings. There were commonalities in some of the headline findings around communication, spiritual needs, autonomy and recording of wishes; although, the studies focussed on different aspects of end‐of‐life care. Table 2 shows full details about the characteristics of the five included studies.

**TABLE 2:** Characteristics of the five included studies in the qualitative evidence synthesis.

Lead author and publication year	Country of research setting	Setting(s) participants recruited from	Participant details			Phenomenon of interest	Recruitment strategy (sampling and eligibility criteria)	Data collection method	Data analysis method	Headline findings
			Number	Age range	Severity^a^					
Bekkema et al. 2016	The Netherlands	Intellectual disability care services, theatre company	33	21–84 (mean 58)	Mild	Dimensions of the care relationship in end‐of‐life care	Purposive sampling. Inclusion criteria were having mild ID and being able to decide about participation and give informed consent. Exclusion criterion was receiving end‐of‐life care.	Group interviews using nominal group technique	Inductive, thematic analysis	These dimensions of the care relationship were established: ‘Ascertain, record and honour wishes’ of the dying person. ‘Being there’ to provide emotional, practical, spiritual and social support
Cithambaram et al. 2020	Northern Ireland	Intellectual disability service covering residential and community settings	11	51–72 (mean 61)	Mild/moderate	Communication and decision‐making at the end of life	Purposive sampling. Inclusion criteria were: having mild or moderate ID; aged over 40; able to articulate and engage in a conversation and give informed consent; and receiving services from the specific provider. Exclusion criteria were being unwilling to give informed consent and suffering bereavement in the last 6 months.	Individual semi‐structured interviews using an interview guide	Constant comparative data analysis	People with intellectual disabilities wished for transparency around life‐limiting conditions and in the context of the rest of their lives. They felt comfortable with this knowledge. It was expressed that a plan of future care should be created to allow professionals to provide care optimally and prevent the occurrence of any ambiguity.
Cithambarm et al. 2021	Northern Ireland	Intellectual disability service covering residential and community settings	11	51–72 (mean 61)	Mild/moderate	Elements of good care at the end of life	Purposive sampling. Inclusion criteria were: having mild or moderate ID; aged over 40; able to articulate and engage in a conversation and give informed consent; and receiving services from the specific provider. Exclusion criteria were being unwilling to give informed consent and suffering bereavement in the last 6 months.	Individual semi‐structured interviews using an interview guide	Constant comparative data analysis	Participants viewed the following elements of end‐of‐life care as essential: effective communication; providing personal care; giving social and spiritual support.
McLaughlin et al. 2014	Northern Ireland	Advocacy group network	17	19–35 = 6; 35–59 = 9; 60+ = 2	*Missing data*	Elements of holistic palliative/end‐of‐life care required and the subsequent education and training needs of healthcare professionals	Purposive sampling. Inclusion criteria were being able to contribute to a group discussion and give valid consent. Exclusion criterion was suffering bereavement in the last 6 months.	Focus groups using a pictorial approach and focused open questions	Content analysis using a recognised framework	Participants felt that people with intellectual disabilities and their family carers require end‐of‐life care that is holistic. This centred around: communication and accessible information, equitable access to palliative care services and the importance of family‐centred care. Findings were in the context of what education should contain for health and social care professionals.
Tuffrey‐Wijne et al. 2013	England	NHS hospitals, Primary Care Trusts and independent organisations	21	*Missing data*	Mild/moderate	Breaking bad news around life‐limiting illness and death	Purposive sampling. Inclusion criterion was having capacity to give informed consent. Participants did not need to have any direct experience of illness or dying. No exclusion criteria were stated.	Focus groups using a range of data collection methods including storytelling, role play and nominal group technique	Content analysis using grounded theory procedures	There were differing views amongst people with intellectual disabilities around whether bad news should be disclosed. The following reasons were given for disclosure: a right to know; ‘knowledge helps the person cope’; and the need for involvement. Preventing distress was the reason given for non‐disclosure.

^a^ Severity of intellectual disability.

### 3.3 Quality Assessment

The quality of reporting across the five included studies was reasonable; however, there were omissions in reporting around certain components and within individual studies, leaving some inconclusive results (see Appendix C). Such omissions are recognised limitations by academics; however, they are still in favour of quality appraisal based on authors' reporting (Carroll et al. 2012).

All five studies were adequately reported according to the four essential criteria in the approach to sensitivity analysis outlined by Carroll et al. (Carroll et al. 2012) (see Appendix D). Thus, none of the studies were privileged based on their quality of reporting and the equivalent contributions of all five studies were examined in the subsequent thematic synthesis (Thomas and Harden 2008).

### 3.4 Data Synthesis

**FIGURE 2:**
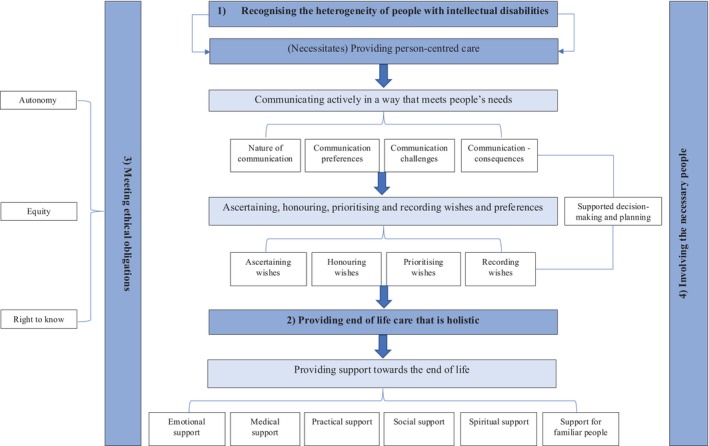
A diagram of the four overarching analytical themes generated through the thematic synthesis and their relationships with the descriptive themes and related sub‐themes *(dark blue = analytical themes, light blue = descriptive themes, white = subthemes)*.

Four overarching analytical themes were generated, and these were supported by three descriptive themes and further related subthemes (see Figure 2). All five of the included studies contributed towards the analytical and descriptive themes and the subsequent recommendations within the discussion.

### 3.5 Theme 1: Recognising the Heterogeneity of People With Intellectual Disabilities

#### 3.5.1 Communicating Actively in a Way That Meets People's Needs

People with intellectual disabilities expressed a need for people to communicate with them towards the end of their life and that the nature of this communication should be ‘active’. This involved: asking people with intellectual disabilities what they want; talking to them; listening to them; and providing opportunities for them to ask questions, seek clarification and share their concerns and feelings. Furthermore, participants expressed that communication should be: empathetic, reassuring and allay their fears. Some study participants also expressed that reminiscing on the past was helpful: “bring up the nice things… that happened”(Bekkema et al. 2016).

The communication needs of people with intellectual disabilities needed to be met in order for active communication to take place. This involved establishing how much a person was able to understand about death and dying, and trying to maximise understanding by making reasonable adjustments, such as the use of non‐verbal communication (Bekkema et al. 2016).

The positive consequences of effective communication towards the end of life were outlined by participants in four of the studies (Bekkema et al. 2016; Cithambaram et al. 2020; Cithambarm et al. 2021; Tuffrey‐Wijne et al. 2013). Some said that effective communication: improved emotional wellbeing, provided opportunities to ask questions, share their concerns, and enabled planning and decision‐making.

Some communication challenges were reflected in the studies. One author (Cithambarm et al. 2021) argued that some people with intellectual disabilities “could not express what they wanted”, and that complex problems prevented the communication of needs. Also, there was sometimes a fear of communication with a dying person by others, resulting in avoiding discussing death or dying. These findings may reflect a wider systemic issue around communication needs being unmet, which could result from barriers such as workforce training issues.

#### 3.5.2 Ascertaining, Honouring, Prioritising and Recording Wishes and Preferences

##### 3.5.2.1 Ascertaining Wishes and Preferences

It is important to ascertain wishes and preferences towards the end of life as this will: “ensure that (a) person feels as good as possible” (Bekkema et al. 2016). Wishes and preferences were expressed around: preferred place of care, funeral wishes and the disclosure of bad news. In three of the studies (Bekkema et al. 2016; Cithambaram et al. 2020; McLaughlin et al. 2014), the importance of ascertaining a person's preferred place of care and funeral wishes was emphasised.

However, there were some differing preferences around disclosure of bad news in four of the studies (Bekkema et al. 2016; Cithambaram et al. 2020; McLaughlin et al. 2014; Tuffrey‐Wijne et al. 2013). Participants in three of these studies (Bekkema et al. 2016; Cithambaram et al. 2020; McLaughlin et al. 2014) solely expressed a preference for disclosure with honest communication and openness, especially from professionals and their families. Some authors (Bekkema et al. 2016; Cithambaram et al. 2020; Tuffrey‐Wijne et al. 2013) justified disclosure by saying it was helpful in enabling people with intellectual disabilities to understand their situation so that they could make decisions, prepare and plan around their future. Also, that disclosure helped to support emotional wellbeing; by reducing anxiety, stress and enabling coping (Cithambaram et al. 2020; Tuffrey‐Wijne et al. 2013). In one of the studies (Tuffrey‐Wijne et al. 2013), however, some participants expressed a preference for non‐disclosure, and the main justification given for this was to prevent distress. These equivocal findings may reflect underpinning ethical tensions around autonomy and a person's ‘right to know’ (see 3.7.1 and 3.7.2), conflicting against internalised perceptions of emotional fragility shaped by overprotective caregivers over time. These tensions may reflect a systemic barrier in providing holistic end‐of‐life care.

##### 3.5.2.2 Recording Wishes and Preferences

The importance of formally recording a person's wishes and preferences towards the end of their life was emphasised in two of the studies (Bekkema et al. 2016; Cithambaram et al. 2020). Examples were given of how this could be done: electronically, ‘on paper’, with ‘wish books’ or using end‐of‐life care plans. The authors of these studies argued that this documentation would facilitate the provision of appropriate care, as everybody involved would understand their role and expectations, and confusion would be removed so that incorrect decisions would be avoided. One of these authors linked the recording of wishes with providing person‐centred care:

“it is clear… that people…receive good care at the end of life when they have a plan of care in place. Participants…believed that recording an individual's wishes would help professionals to provide person‐centred care.” (Cithambaram et al. 2020)

##### 3.5.2.3 Honouring and Prioritising Wishes and Preferences

There was consensus amongst participants in two of the studies (Bekkema et al. 2016; Cithambaram et al. 2020) that a person's wishes and preferences should be prioritised:

“you should listen to (their) wishes. And meet (their) wishes. It is about (them). What (they) want. And how (they) want it.” (Cithambaram et al. 2020)

However, perspectives diverged within the same two studies around whether ascertaining and recording wishes and preferences ensured that they would be honoured. Some participants vigorously expressed mistrust around whether wishes and preferences would be honoured and illustrated instances where this had not happened.

### 3.6 Theme 2: Providing End‐of‐Life Care That Is Holistic

#### 3.6.1 Support Towards the End of Life

Participants across the studies considered the different types of support that contribute towards providing holistic end‐of‐life care.

##### 3.6.1.1 Emotional Support

Two studies highlighted the importance of those surrounding a person with intellectual disabilities towards the end of life demonstrating empathy towards them by saying comforting words, encouraging or consoling them (Bekkema et al. 2016; Tuffrey‐Wijne et al. 2013). In two studies, participants highlighted that people with intellectual disabilities should be enabled to think, feel and experience positive things towards the end of their life; whether this was feeling “joy and peace” or “experienc(ing) fun and pleasure” (Bekkema et al. 2016; Cithambarm et al. 2021).

##### 3.6.1.2 Medical Support

Two studies referred to the need for medical support with tablets, fluids and tube‐feeding, and highlighted the importance of medical care being ‘evidence‐based’ (Cithambaram et al. 2020; Cithambarm et al. 2021).

##### 3.6.1.3 Practical Support

There were concerns in three of the studies that people with intellectual disabilities would lose their independence and rely on others for practical support towards the end of their lives (Bekkema et al. 2016; Cithambarm et al. 2021; McLaughlin et al. 2014). Participants in these studies gave examples where practical support might be needed, including washing, bathing, feeding and support with household chores (Bekkema et al. 2016; Cithambarm et al. 2021; McLaughlin et al. 2014). Supporting these needs could enable people with intellectual disabilities to participate in everyday activities despite their terminal illness; this participation was important to them (Cithambarm et al. 2021).

##### 3.6.1.4 Social Support

In three of the studies (Bekkema et al. 2016; Cithambarm et al. 2021; McLaughlin et al. 2014), participants expressed the importance of others ‘being there’ for a person with intellectual disabilities towards the end of life. Participants expressed the need for meaningful companionship, love, physical presence and connection: “the simple act of physically touching, such as holding hands, provides a close personal message that makes people feel connected when they are nearing death” (Cithambarm et al. 2021). Such social support was linked with emotional support, providing: comfort, reassurance, self‐identify, positivity, enjoyment and happiness. However, in one of these studies participants were concerned that social support would be missing due to “fear(s) of staying and making conversation” (Cithambarm et al. 2021). In this same study, the author argued that social support should be balanced with time alone. However, there was divergence around this in the other two studies (Bekkema et al. 2016; McLaughlin et al. 2014), where the importance of “not letting the person be alone” was instead emphasised (Bekkema et al. 2016).

##### 3.6.1.5 Spiritual Support

Participants across three studies (Bekkema et al. 2016; Cithambarm et al. 2021; McLaughlin et al. 2014) discussed the importance of providing spiritual support to a person with intellectual disabilities towards the end of their life. It was argued that spiritual support is equal to other components of care and could help a person by: reminiscing about their past; coming to terms with their present; reconciling with themselves and others (including God where applicable); discussing and reducing fears; providing emotional support; and ultimately preparing them for the end of their life.

In two of the studies (Bekkema et al. 2016; Cithambarm et al. 2021), participants expressed the importance of religious and faith support. This was through religious caregivers such as pastors, chaplains and priests. In one of these studies (Cithambarm et al. 2021), participants felt that prayers and blessings would spiritually and psychologically comfort a person at the end of their life.

##### 3.6.1.6 Support for Familiar People

In two studies (Cithambaram et al. 2020; McLaughlin et al. 2014), participants discussed the need to support familiar people around the terminally ill person, and that this should include bereavement support.

### 3.7 Theme 3: Meeting Ethical Obligations

#### 3.7.1 Autonomy

The importance of respecting a person's autonomy towards the end of life was explicitly emphasised in three studies, as illustrated by this participant: “you should listen to her wishes. And meet her wishes. It is about her. What she wants. And how she wants it. That's the last thing you can do for her” (Bekkema et al. 2016). However, there were concerns from another participant in the same study that autonomy was not being respected:

“people don't want us to think for ourselves and make decisions. They want to arrange everything themselves, they think: we know best, and we will decide…that's their attitude.” (Bekkema et al. 2016)

#### 3.7.2 Right to Know

A person's ‘right to know’ about their care contributes to ensuring that their autonomy is respected:

“people with intellectual disabilities expressed that they had a right to know what was happening to them… (and) can decide what they want and how much they want to know.” (Cithambaram et al. 2020)

Some participants in two of the studies used this justification around disclosure of bad news to a person with intellectual disabilities (Cithambaram et al. 2020; Tuffrey‐Wijne et al. 2013). In one of these studies, it was argued that the communication resulting from a person's right to know, enabled choices and decisions to be made by a person with intellectual disabilities towards the end of their life.

#### 3.7.3 Equity

In two of the studies (Cithambarm et al. 2021; McLaughlin et al. 2014), the need for equitable end‐of‐life care was highlighted. To enable this, one participant highlighted that: “we should be giving them more care than everybody else” (Cithambarm et al. 2021).

### 3.8 Theme 4: Involving the Necessary People

Across the five studies, there was were roles for ‘necessary people’ in: communication; ascertaining, honouring, prioritising and recording wishes and preferences; and providing holistic support to people with intellectual disabilities towards the end of life.

Firstly, around communication, participants across the five studies outlined the role for professionals, such as doctors, nurses and carers in: breaking bad news; explaining around an illness; informing around care options; supporting decision‐making and planning; and providing opportunities for people to ask questions, seek clarification and share their concerns. There were also roles for familiar people, such as family, friends, co‐habitants and peers in: supporting with communication needs, having open conversations and breaking bad news. This support of familiar people was felt to be particularly valuable when a person was non‐verbal (Bekkema et al. 2016).

Secondly, across four of the studies there were differing perspectives around responsibilities for ascertaining, honouring, prioritising and recording wishes and preferences. There were roles for both professionals and family members in ascertaining the preferred place of care, practical and medical needs of a person with intellectual disabilities (Bekkema et al. 2016). There was also consensus that spiritual caregivers or religious leaders should ascertain spiritual support needs (Bekkema et al. 2016; Cithambarm et al. 2021). However, there were differing perspectives around who should disclose bad news. In three studies (Cithambaram et al. 2020; McLaughlin et al. 2014; Tuffrey‐Wijne et al. 2013), participants felt that this was the responsibility of professionals. Although, some also felt that there was a role for the family to disclose if professionals were not understood (McLaughlin et al. 2014). The responsibility for recording and honouring wishes was levelled at professionals alone in two of the studies (Bekkema et al. 2016; Cithambaram et al. 2020). Importantly, in the same two studies the responsibility for everybody in prioritising wishes and preferences was emphasised: “people should make sure that the person with ID is in control, that their wishes take priority” (Bekkema et al. 2016).

Finally, there were differing views about who should provide holistic support. This varied depending on the type of support needed and the availability and skills of the supporter. In four of the studies (Bekkema et al. 2016; Cithambaram et al. 2020; Cithambarm et al. 2021; McLaughlin et al. 2014), participants felt that there was a role for both professionals and familiar people in providing practical and social support towards the end of life. However, some participants also outlined a role for professionals in: providing medical support; transferring skills or upskilling others to provide practical support; and in providing social support for familiar people (Cithambaram et al. 2020; Cithambarm et al. 2021). There was also a role for familiar people in providing emotional support, with one author emphasising that: “(familiar people) are the best people to provide (it)” (Bekkema et al. 2016). In three of the studies (Bekkema et al. 2016; Cithambarm et al. 2021; McLaughlin et al. 2014), participants held similar views around the vital role of familiar people in providing social support. However, there were challenges highlighted for both professionals and familiar people in meeting a person's support needs. This is because these needs may be extensive, with some people requiring “close supervision”, “vigilant monitoring” and “continuous care” up to “24 h a day” (Bekkema et al. 2016; Cithambarm et al. 2021). There were also concerns around the availability of ‘necessary people’. This may have been due to a lack of: family contact, family proximity, professional staffing, willingness, or ability to provide support (Bekkema et al. 2016; Cithambarm et al. 2021). It was felt that responsibility for providing spiritual support lay with spiritual caregivers and religious leaders (Bekkema et al. 2016; Cithambarm et al. 2021; McLaughlin et al. 2014). However, participants articulated a universal role that “anyone could do” around prayer (Cithambarm et al. 2021).

## 4 Discussion

### 4.1 Summary of Evidence

Viewed from their own perspectives, these interrelated themes demonstrate what contributes to the optimal end‐of‐life care of people with intellectual disabilities (see Figure 2). Care needs to be person‐centred and individualised rather than treating people with intellectual disabilities as a homogenous group (Theme 1). Person‐centred care relies on active communication with a person with intellectual disabilities and it should meet their needs. This is necessary to enable their wishes and preferences to be ascertained, honoured, prioritised and recorded towards the end of life. Establishing these wishes and preferences is a prerequisite for providing holistic end‐of‐life care (Theme 2). Holistic care must meet a person's emotional, practical, social and spiritual needs as well as their medical needs. In addition, it is important that ‘familiar people’ support the person as part of this care.

The ethical and legal principles of: autonomy, equity and a person's ‘right to know’ (Theme 3) underpin all of these themes. Furthermore, to facilitate end‐of‐life care that is both person‐centred and holistic, there needs to be involvement of the ‘necessary people’ in a person's care (Theme 4). Necessary people will be identified according to the needs, wishes and preferences of people with intellectual disabilities, as well as their availability and skills.

### 4.2 Comparison With Existing Literature

There is much consensus between this qualitative evidence synthesis and the existing literature; however, there are some areas of divergence.

This synthesis found that to provide person‐centred care, communication with people with intellectual disabilities needs to be ‘active’. The need for active communication to facilitate understanding of wishes, preferences and needs, along with supporting decision‐making and planning was reflected in international palliative care standards (African Palliative Care Association 2011) and in the National End Of Life Care Programme (NEOLCP) pathway (NHS National End of Life Care Programme 2017) in the UK. The need to overcome challenges in communication, including fear of communication, has too been outlined within international norms (European Association for Palliative Care 2015) and standards (African Palliative Care Association 2011), and in previous studies (Tuffrey‐Wijne et al. 2009). The importance of ascertaining a person's wishes and preferences towards the end of life is emphasised in international norms (European Association for Palliative Care 2015), standards (African Palliative Care Association 2011), position statements (American Association on Intellectual and Developmental Disabilties 2020); and is reflected through several steps of the NEOLCP pathway (NHS National End of Life Care Programme 2017) in the UK. The differing preferences of participants around disclosure of bad news in this synthesis were reflective of previous findings in the literature (Cithambarm et al. 2021; Tuffrey‐Wijne et al. 2009, 2010). These findings are then extended within this synthesis, international norms (European Association for Palliative Care 2015) and a international position statement (American Association on Intellectual and Developmental Disabilties 2020), by expressing the importance of recording, prioritising and honouring these wishes and preferences too.

Additionally, there was consensus around the need to support a person holistically, including supporting those familiar to them. Within this synthesis, international norms (European Association for Palliative Care 2015) and standards (African Palliative Care Association 2011). This is also reflected through the steps of the NEOLCP pathway (NHS National End of Life Care Programme 2017) and ambitions by the Palliative Care of People with Learning Disabilities (PCPLD) Network (Palliative Care For People With Learning Disabilities Network 2017) within the UK. Although the international African palliative care standards go further in considering cultural support and culturally competent care as part of wider holistic support.

Furthermore, the three ethical principles underpinning optimal end‐of‐life care in this synthesis were reflected in ambitions within the UK (Palliative Care For People With Learning Disabilities Network 2017). Internationally, the principle around equity of access was stated widely in guidance, norms, standards and position statements (African Palliative Care Association 2011; American Association on Intellectual and Developmental Disabilties 2020; European Association for Palliative Care 2015; World Health Organisation 2016). The principle around a person's ‘right to know’ was also outlined in the European consensus norms and American position statement, however, these publications went further in exploring the right to recognition of the value of life. Also, despite different legal frameworks internationally, the European Association for Palliative Care advocated for the assumption of capacity until proven otherwise (European Association for Palliative Care 2015). The final principle around autonomy, and respecting this, was also explicitly stated in the African palliative care standards. However, within this synthesis there were concerns in one of the studies (Bekkema et al. 2016) around not respecting a person's autonomy in favour of the views of others. These concerns are supported by the findings of previous literature (Bekkema et al. 2014a, 2014b; Tuffrey‐Wijne and McEnhill 2008; Wagemans et al. 2010). Finally, both this synthesis and the European Association for Palliative Care through its consensus norms (European Association for Palliative Care 2015), advocated for the importance of involving the necessary people or the people ‘who matter’.

### 4.3 Limitations

There are four key limitations to this review. Firstly, all studies were conducted in the UK or the Netherlands, so the extent to which findings can be extrapolated to different countries may be limited. Secondly, people with severe to profound intellectual disabilities were not recruited in any of the studies and so their views are not included in this synthesis. Third, none of the studies included participants who were in receipt of end‐of‐life care themselves, nor did they need to have direct experience of witnessing this care for others. This was likely to be due to the ethical ramifications of recruitment in this context; however, it might limit the extent to which the views of participants are representative of people with intellectual disabilities who are in receipt of end‐of‐life care. Finally, this review was originally submitted as a Master's dissertation and as such it was limited in the extent to which a second reviewer could be involved in quality assessment, data extraction and data synthesis. This may limit the reliability of these stages; however, we attempted to account for this by discussing each stage in detail during supervisory meetings.

### 4.4 Recommendations

Practitioners should ensure that when caring for a person with intellectual disabilities at the end of their life they: communicate actively with them; meet their holistic support needs; respect their wishes and preferences; meet relevant ethical obligations and involve the necessary people in their care.

Policy‐makers should ensure that their policies guide the delivery of this optimal end‐of‐life care and that professionals together with other supporters are available, competent and empowered to provide such care.

Researchers should conduct further research about the way in which end‐of‐life care can be tailored to meet the needs of people with intellectual disabilities. In particular, they should strive to represent the diverse contexts, experiences and capabilities within the population of people with learning disabilities, to better understand how to provide high‐quality end‐of‐life care to this underserved group. Tuffrey‐Wijne et al. (2009) argued that ethnographic methods could capture the perspectives of people with more severe intellectual disability, and that this would be a valuable addition to the literature.

## 5 Conclusion

From the perspectives of people with intellectual disabilities, optimal end‐of‐life care must be person‐centred and thereby recognise the heterogeneity of people with intellectual disabilities. This person‐centred care should be driven by active communication to identify an individual's wishes, preferences, and holistic support needs. Supporting someone holistically means supporting them emotionally, medically, practically, socially and spiritually. The ethical principles of autonomy, equity and a person's ‘right to know’ underpin this person‐centred and holistic care. Ultimately, this care should be delivered by the ‘necessary’ people, as determined by the needs, wishes and preferences of the person with intellectual disabilities, as well as by their availability and skills.

## Conflicts of Interest

The authors declare no conflicts of interest.

## Data Availability

The authors have nothing to report.

## Appendix A Sample Search Strategy in Ovid MEDLINE: 1946 to 11 July 2022

1 exp Learning Disabilities/23098

2 (Learning adj2 (disorder* or disab* or defici* or difficult* or impair*)).tw.24523

3 exp Intellectual Disability/103083

4 (Intellectual* adj2 (disorder* or disab* or defici* or difficult* or impair*)).tw.25298

5 exp Developmental Disabilities/21985

6 (Developmental* adj2 (disorder* or disab* or defici* or difficult* or impair*)).tw.22977

7 Cognition Disorders/66178

8 (Cognit* adj2 (disorder* or disab* or defici* or difficult* or impair*)).tw.121477

9 Special need*.tw.4755

10 Special education*.tw.4243

11 Mental* deficien*.tw.1977

12 Mental* retard*.tw.33519

13 exp Congenital Abnormalities/639014

14 exp Brain Damage, Chronic/40026

15 exp Hypoxia, Brain/13990

16 exp Down Syndrome/25869

17 (Down* syndrome or trisomy 21).mp. or trisomy.tw. [mp = title, book title, abstract, original title, name of substance word, subject heading word, floating sub‐heading word, keyword heading word, organism supplementary concept word, protocol supplementary concept word, rare disease supplementary concept word, unique identifier, synonyms]45,123.

18 (congenital adj2 (defect* or disorder* or condition* or anomal* or abnormalit*)).tw.60160

19 (genetic adj2 (defect* or disorder* or condition* or anomal* or abnormalit* or disease*)).tw.68676

20 (chromosomal adj2 (defect* or disorder* or condition* or anomal* or abnormalit* or disease*)).tw.17672

21 (birth adj3 (defect* or complication* or abnormalit*)).tw.14586

22 (fetal adj2 (defect* or disorder* or condition* or anomal* or abnormalit* or disease* or hypox*)).tw.12568

23 (brain adj3 (damage* or hypox* or isch?em* or injury)).tw.115923

24 (cerebral adj3 (damage* or hypox* or isch?em* or injury or palsy)).tw.70739

25 1 or 2 or 3 or 4 or 5 or 6 or 7 or 8 or 9 or 10 or 11 or 12351320

26 13 or 14 or 15 or 16 or 17 or 18 or 19 or 20 or 21 or 22 or 23 or 24952471

27 25 or 261220033

28 exp Palliative Care/60973

29 exp Terminal Care/55796

30 exp Advance Care Planning/10826

31 exp Death/160504

32 end of life care.tw.11464

33 dying.tw.38420

34 die.tw.68965

35 death*.tw.950658

36 exp Bereavement/14740

37 (terminal* adj2 (care* or condition* or ill* or diagno*phase*)).tw.10124

38 (life limiting* adj3 (condition* or ill* or diagno* or phase*)).tw.1698

39 (last moment* or last hour* or last day* or last week* or last month* or last year*).tw.41651

40 (final moment* or final hour* or final day* or final week* or final month* or final year*).tw.7180

41 exp Hospices/5552

42 hospice*.tw.14103

43 (((“semi‐structured” or semistructured or unstructured or informal or “in‐depth” or indepth or “face‐to‐face” or structured or guide) adj2 (interview* or discussion* or questionnaire*)) or (focus group* or qualitative or ethnograph* or fieldwork or “field work” or “key informant”)).tw,kw. or interviews as topic/or focus groups/or narration/or qualitative research/476495

44 28 or 29 or 30 or 31 or 32 or 33 or 34 or 35 or 36 or 37 or 38 or 39 or 40 or 41 or 42 1262112

45 27 and 43 and 44 896

## Appendix B Caroll et al. Reporting Assessment Checklist

**Table:**

Criteria	Categorization	Definition
1. The question and study design	Yes	If the choice of study design was given and explained
	No	If article does not specify question and study design
2. The selection of participants	Yes	If the selection of participants is described explicitly as, e.g., purposive, convenience, theoretical and so forth
	No	If only details of participants are given
3. Methods of data collection	Yes	If details of the data collection method are given, e.g., piloting, topic guides for interviews, number of items in a survey, use of open or closed items, validation and so forth
	No	If just only states focus group, interview or questionnaire
4. Methods of analysis	Yes	If details of analysis method are given, e.g., transcription and form of analysis (with reference to or full description of method), validation tests and so forth
	No	If only states content analysis or that data were analysed

## Appendix C Quality Appraisal Results for the Five Included Studies

**Table:**

Study details	Component of the CASP Qualitative Appraisal Checklist								
	Clear aims	Methodology	Study design	Recruitment strategy	Data collection	Relationships: bias	Ethical issues	Data analysis	Clear findings
Bekkema et al. 2016	✓	✓	—	✓	—	—	✓	✓	✓
Cithambaram et al. 2020	✓	✓	—	✓	—	—	✓	✓	✓
Cithambarm et al. 2021	✓	✓	—	✓	✓	—	✓	✓	✓
McLaughlin et al. 2014	✓	✓	✓	—	✓	—	—	—	—
Tuffrey‐Wijne et al. 2013	✓	✓	—	✓	✓	—	—	✓	✓

*Note:* A tick indicates yes and a dash (—) indicates that this component was unclear.

## Appendix D If Included Studies Were Adequately or Inadequately Reported Using the Four Essential Quality Assessment Criteria Described by Caroll et al. Is Shown

**Table:**

Study details	Four essential quality assessment criteria				Adequately or inadequately reported
	Study design	Recruitment strategy	Data collection	Data analysis	
Bekkema et al. 2016	—	✓	—	✓	Adequately
Cithambaram et al. 2020	—	✓	—	✓	Adequately
Cithambarm et al. 2021	—	✓	✓	✓	Adequately
McLaughlin et al. 2014	✓	—	✓	—	Adequately
Tuffrey‐Wijne et al. 2013	—	✓	✓	✓	Adequately

*Note*: A tick indicates yes and a dash (—) indicates that this component was unclear.
